# Galectin-3 Is a Potential Mediator for Atherosclerosis

**DOI:** 10.1155/2020/5284728

**Published:** 2020-02-14

**Authors:** Ziyu Gao, Zhongni Liu, Rui Wang, Yinghong Zheng, Hong Li, Liming Yang

**Affiliations:** ^1^State Key Laboratory of Cardiovascular Disease, Fuwai Hospital, National Center for Cardiovascular Diseases, Beijing 100037, China; ^2^Department of Pathophysiology, Key Laboratory of Cardiovascular Pathophysiology, Harbin Medical University, Harbin 150081, China; ^3^Yangpu Hospital, Tongji University, Shanghai 200090, China

## Abstract

Atherosclerosis is a multifactorial chronic inflammatory arterial disease forming the pathological basis of many cardiovascular diseases such as coronary heart disease, heart failure, and stroke. Numerous studies have implicated inflammation as a key player in the initiation and progression of atherosclerosis. Galectin-3 (Gal-3) is a 30 kDa *β*-galactose, highly conserved and widely distributed intracellularly and extracellularly. Gal-3 has been demonstrated in recent years to be a novel inflammatory factor participating in the process of intravascular inflammation, lipid endocytosis, macrophage activation, cellular proliferation, monocyte chemotaxis, and cell adhesion. This review focuses on the role of Gal-3 in atherosclerosis and the mechanism involved and several classical Gal-3 agonists and antagonists in the current studies.

## 1. Introduction

Atherosclerosis has become the prelude and the major manifestation of ischemic coronary-cerebrovascular disease such as ischemic heart disease and stroke. In the cohort of patients with ischemic stroke, the prevalence of atherosclerosis is increasing worldwide especially in Asian populations [1]. Atherosclerosis is a chronic inflammatory disease characterized by excessive accumulation of lipoprotein in macrophage, monocyte chemoattraction in vascular lesion, and the infiltration of vascular smooth muscle cells (VSMC) into the subendothelial space. Accumulating studies have indicated that inflammation plays an important role in the initiation and progression of atherosclerosis [2, 3].

Galectin-3 (Gal-3) is currently regarded as a potential cardiovascular inflammatory biomarker. It is a 29-35 kDa highly conserved *β*-galactoside-binding lectin and has received widespread interest in cardiovascular disease in recent decades. Gal-3 has been identified as a proinflammatory molecule that functions to drive the inflammatory response and oxidative stress. In addition, Gal-3 has an impact on the progress of atherosclerosis including endothelial dysfunction, lipid endocytosis, and VSMC migration. The role of Gal-3 in the cardiovascular area has been summarized by several review articles. However, previous reviews mainly focused on the association between Gal-3 and heart failure [4–6], and the influence of Gal-3 on atherosclerosis has not been carefully summarized. Up to now, extensive research has been carried out in the basic and epidemiological areas investigating the influence of Gal-3 on atherosclerosis. This review therefore summarizes the available research evidence on the effect of Gal-3 on atherosclerosis and the application of Gal-3 agonists and antagonists, with the aim of providing a better overview of Gal-3 as a new biomarker and contributor for atherosclerosis.

## 2. Galectin-3

### 2.1. Galectin-3 and Its Biochemical Activities

Among the galectin members, Gal-3 is the only member of vertebrate chimera-type galectin that contains a C-terminal carbohydrate recognition domain (CRD) and an N-terminal peptide [7]. The N-terminal peptide contains the first 12 amino acids and an internal repeating domain. The CRD contains approximately 130 amino acids and specifically recognizes and binds to glycoprotein oligosaccharides expressed on the cell surface, within cells or in the extracellular matrix. Gal-3 proteins usually exist as monomers but can also form pentamers via N-terminal domain association when the concentration of Gal-3 monomers is high [8–10]. Gal-3 is coded by LGALS3 in the human genome and synthesized in the cytoplasm, then transported to the nucleus, other organelles, or secreted into the extracellular space. As Gal-3 lacks a signal sequence for insertion into the endoplasmic reticulum, Gal-3 can be secreted into the extracellular space via a nonclassical pathway [11, 12]. Gal-3 can be detected in a wide range of tissues and cells including skin, brain, intestinal tract, liver, and various cancer cells [13]. In addition, several studies have suggested that activated macrophage, monocyte, neutrophil, and mast cell also express Gal-3 [12, 14]. With its expression significantly and rapidly induced under diseased conditions, Gal-3 has received great interest over other galectins for its role in a variety of diseases including cancer, diabetes, and heart disease. As an important inflammatory biomarker, Gal-3 can promote the secretion of other proinflammatory factors like tumor necrosis factor-*α* (TNF-*α*) and interleukin-6 (IL-6) through activation of macrophages in a dose-dependent manner [15]. Gal-3 also participates in the progression of lipid endocytosis, cell apoptosis, cell differentiation, cell adhesion, and tumor metastasis [16–18].

### 2.2. Gal-3 and Inflammation

Gal-3 is a central regulator of critical processes under the setting of acute and chronic inflammation. Gal-3 is involved in the process of acute inflammatory response including chemoattraction of monocytes/macrophages [19], neutrophil clearance [20], opsonization of apoptotic neutrophils [21], and mast cell degranulation [22]. Through interacting with nucleotide oligomerization domain-like receptor protein 3 (NLRP3), intracellular Gal-3 enhanced the effects of H5N1 infection by promoting host inflammatory responses and regulating interleukin-1 beta (IL-1*β*) production by macrophages. Compared to infected WT mice, infected Gal-3 knockout mice exhibited less inflammation in the lungs and reduced IL-1*β* levels in bronchoalveolar lavage fluid [23]. Chronic inflammation usually accompanied with fibrosis, loss of tissue structure, and subsequent organ failure is a heavy healthcare burden worldwide. Fibroblasts that represent key cells in the initiation and perpetuation of tissue fibrogenesis can promote inflammation by secreting inflammatory factors such as TNF-*α*, IL-6, and chemokines including C-X-C motif chemokine 8 (CXCL8), C-C chemokine ligand 2 (CCL2), C-C chemokine ligand 3 (CCL3), and C-C chemokine ligand 5 (CCL5) upon the activation of Gal-3 [24]. The expression of CCL2, CCL5, and CXCL8 stimulated by Gal-3 can attract monocyte and macrophage to the vascular lesion and plaque, especially in the core of lipid accumulation [15]. Gal-3 can bind with integrin in the cell surface to promote the adhesion between neutrophil and vascular endothelial cell through modifying cell-cell interaction [25]. Gal-3 has been identified as a critical molecule that mediates immunological functions in multiple immune cells, such as dendritic cells (DCs), B cells, and macrophages. Gal-3 inhibition downregulated expression of IL-6, IL-1*β*, and IL-23 p19, while it upregulated IL-10 and IL-12 p35 in toll-like receptor/NOD-like receptor- (TLR/NLR-) stimulated human monocyte-derived DCs, which inhibited subsequent Th17 and Th2 development. This finding indicated that intracellular Gal-3 acted as a cytokine hub of human DCs in responding to innate immunity signals [26]. Moreover, B cells with restrained endogenous Gal-3 expression skewed the balance toward plasma cell differentiation, which resulted in increased immunoglobulin production and parasite clearance during T. cruzi infection, providing evidence of a novel role for Gal-3 as an intracellular mediator of B cell survival and differentiation [27]. Disruption of the Gal-3 expression restrained IL-4/IL-13-induced alternative macrophage activation in recruited peritoneal macrophages *in vivo* without affecting IFN-*γ*/LPS-induced classical activation or IL-10-induced deactivation [28].

### 2.3. Gal-3 and Oxidative Stress

The relationship between Gal-3 and oxidative stress has been demonstrated *in vitro*, such that the treatment of monocytes with phorbol myristate acetate, a nicotinamide adenine dinucleotide phosphate (NADPH) oxidase-dependent inducer of reactive oxygen species, produced an increase in Gal-3 mRNA and protein expression [29]. Gal-3 stimulated the hyperoxide secretion from neutrophils through activation of NADPH II [30]. Excess hyperoxide led to an increased expression of cell surface glycoprotein and enhanced the oxidative stress induced by ischemia or reperfusion damage to further exacerbate vascular lesion [31]. Gal-3 was shown to induce oxidative stress through the release of O_2_^−^ in cultured mast cells, an effect that was blocked by the antioxidant enzyme superoxide dismutase [32]. A study revealed that plasma Gal-3 concentration was increased in peripheral artery disease patients and correlated with F2-isoprostanes, the serologic marker of oxidative stress [33].

## 3. Gal-3 and Atherosclerosis

### 3.1. Gal-3 Level Increased in Atherosclerotic Vessels

**Figure 1:**
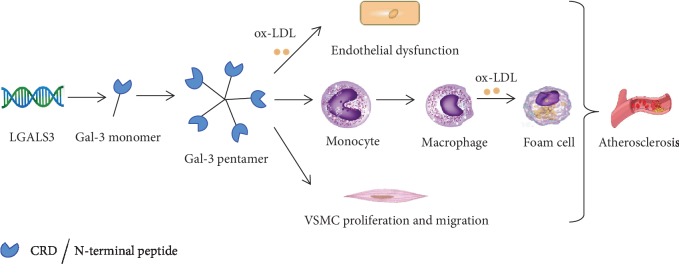
Gal-3 and its effect on different types of cells related to atherosclerosis. Gal-3: galectin-3; ox-LDL: oxidized low-density lipoprotein; VSMC: vascular smooth muscle cells; CRD: C-terminal carbohydrate recognition domain.

Atherosclerosis is considered to be a complex inflammatory process that involves various inflammatory markers. In recent years, the relationship between Gal-3 and atherosclerosis has been investigated by substantial experimental studies. The level of Gal-3 was demonstrated to develop in human atherosclerosis plaque and some animal models, especially hypercholesterolemic rabbits and Apolipoprotein-E knockout (ApoE^−/−^) mice with vascular stenosis. A study compared the Gal-3 level of aortic tissue between ApoE^−/−^ mice on a high-fat diet and wild-type control and found that Gal-3 mRNA and protein levels in ApoE^−/−^ mice were elevated 16.3 and 12.2 times than those of wild type, respectively. Moreover, the expression of Gal-3 increased in unstable plaque compared with stable regions from the same patient at both mRNA and protein levels, and Gal-3-treated human macrophages induced an 11-fold increase in human monocyte chemotaxis [15]. Endothelial cells, macrophages, and VSMC are the most important types of cells involved in the development of atherosclerosis, and Gal-3 can affect all these kinds of cells (Figure 1).

### 3.2. Gal-3 and Endothelial Dysfunction

Endothelial cells (ECs) play a crucial role in maintaining vascular homeostasis in response to various stimuli. Under conditions of chronic inflammation, sustained activation of ECs by inflammatory stimuli causes alterations in normal endothelial function, resulting in endothelial dysfunction which has been considered to be the basis and initial step of atherosclerosis, the most common cause of cardiovascular diseases [34, 35]. Several studies have shown that oxidized low-density lipoprotein (ox-LDL) induced endothelial cell injury by changing proinflammatory gene expression [36, 37]. Accumulating evidence has demonstrated that Gal-3 aggravated ox-LDL-mediated endothelial injury by inducing inflammation [38, 39]. Inhibition of Gal-3 abrogated cigarette smoke extract- (CSE-) induced autophagy and dysfunction of endothelial progenitor cells (EPCs) which have the potential to repair damaged blood vessels and promote angiogenesis [40]. And it was indicated that further progression of endothelial dysfunction increased the atherosclerotic plaque burden and Gal-3 staining in NADPH oxidase 4/low-density lipoprotein receptor knockout (Nox4^−/−^/Ldlr^−/−^) mice compared with Ldlr^−/−^ mice [41]. However, Gal-3 deficiency led to exacerbated metabolic derangement and endothelial dysfunction in diabetic mice. And coagulation activity was enhanced suggesting a protective role for Gal-3 against thrombosis [42].

### 3.3. Gal-3 in Macrophage Differentiation and Foam Cell Formation

Ox-LDL induces endothelial dysfunction with focal inflammation which in turn causes increased expression of atherogenic signaling molecules that promote the adhesion of monocytes to the arterial endothelium and their penetration into the intima. Some studies have indicated that the synthesis and expression of Gal-3 are associated with differentiation and activation of macrophage. Liu et al. indicated that Gal-3 can be expressed on the surface of normal human peripheral blood monocytes. This finding indicated that Gal-3 levels increased significantly as monocytes differentiated into macrophages *in vitro*, and the secretion of Gal-3 by monocytes was regulated by lipopolysaccharide or interferon-*γ* [43]. Meanwhile, it has been demonstrated that human macrophage was the main origin of Gal-3 in both mRNA and protein levels [15]. Kim et al. indicated that Gal-3 expression in macrophage was signaled by Ras/MAP kinase pathway and that it can be upregulated by modified lipoprotein [44]. Thus, the elevated Gal-3 in atherosclerosis is associated with macrophage. One study demonstrated that Gal-3 is mainly distributed in macrophages and foam cells which are major components of atherosclerosis plaques, but not in VSMC, and Gal-3 expression increased with the plaque severity [45]. Similarly, another study found that the intraplaque Gal-3 expression levels were proportionally elevated as the degree of plaque extent and inflammation increased [46]. This finding showed that Gal-3 was heavily and exclusively accumulated in intimal plaques and that Gal-3 distribution was colocalized with plaque macrophages' distribution. The process of differentiated macrophages absorbing ox-LDL and transforming into foam cells has a profound association with Gal-3. Zhu et al. demonstrated that Gal-3 promoted lipoprotein uptake of foam cells to exacerbate atherosclerosis [47]. Moreover, the effect of Gal-3 on cardiac metabolic disturbance associated with obesity has been investigated. Marín-Royo et al. found that Gal-3 inhibition attenuated the consequences of cardiac lipotoxicity induced by high-fat diet *in vivo* [16]. Therefore, Gal-3 might exacerbate atherosclerosis plaque through promoting endocytosis of lipoprotein and disturbing lipid metabolism.

### 3.4. Gal-3 Stimulates VSMC Proliferation and Migration

**Figure 2:**
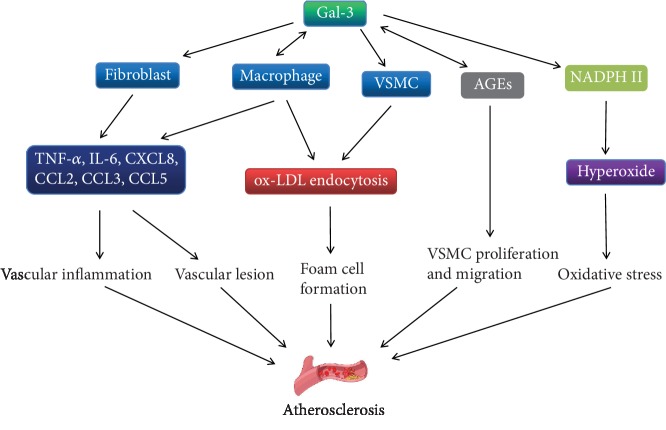
Diagram depicting the mechanisms by which Gal-3 promotes formation of atherosclerosis. Gal-3: galectin-3; TNF-*α*: tumor necrosis factor-*α*; IL-6: interleukin-6; CXCL8: C-X-C motif chemokine 8; CCL2: C-C chemokine ligand 2; CCL3: C-C chemokine ligand 3; CCL5: C-C chemokine ligand 5; ox-LDL: oxidized low-density lipoprotein; VSMC: vascular smooth muscle cells; AGEs: advanced glycation end products; NADPH II: nicotinamide-adenine dinucleotide phosphate II.

Proliferation and migration of VSMC are important processes of atherosclerosis. Although VSMC are not the major origin of Gal-3 in circulating blood, the influence of Gal-3 on VSMC stimulates atherosclerosis. Tian et al. reported that Gal-3 expression increased in the phenotypic transformed VSMC treated with ox-LDL. Small interfering RNA silencing or knockdown of Gal-3 inhibited the phenotypic transformation and migration of VSMC [48], and another study of Tian et al. particularly indicated that exogenous Gal-3 promoted human VSMC proliferation and migration through the activation of canonical Wnt/*β*-catenin signaling pathway [49]. Menini et al. demonstrated that the effect of Gal-3 on VSMC within atherosclerotic plaque was related to the receptor for advanced glycation end products (RAGE) [50]. Gal-3 has been identified as an advanced glycation end product (AGE) receptor. Extracellular AGEs and its modified protein can bind with Gal-3 on the cell surface to form a protein complex and then attenuate the adhesion between VSMC and matrix glycoprotein. This attenuation of adhesion stimulates the proliferation and migration of VSMC for atherosclerosis exacerbation [47, 51]. These studies provide future directions for research on the treatment of atherosclerosis targeting on VSMC. Thus, as illustrated in Figure 2, Gal-3 can lead to atherosclerosis through inflammatory activation, vascular lesion, lipid endocytosis, VSMC migration, and oxidative stress.

## 4. Genetic Studies of Gal-3 in Atherosclerosis

Previous genetic studies of Gal-3 and atherosclerosis mainly focused on the comparison between Apolipoprotein-E/Gal-3 double knockout (ApoE^−/−^/gal-3^−/−^) mice and ApoE^−/−^ mice. Mackinnon et al. indicated that the atherosclerosis plaque area of ApoE^−/−^/gal-3^−/−^ mice with a high-cholesterol diet was significantly smaller than that of ApoE^−/−^ mice. Compared with ApoE^−/−^ mice, the aortic plaque bulk of ApoE^−/−^/gal-3^−/−^ mice with a 12-week and 20-week high-cholesterol diet reduced by 35% and 40%, respectively [52]. Nachtigal et al. compared the severity of periaortic vascular adventitia inflammation between ApoE^−/−^/gal-3^−/−^ mice and ApoE^−/−^ mice and found that aortic atherosclerosis plaques increased and vascular adventitia inflammation was aggravated in ApoE^−/−^ mice as the age increased, but this phenomenon did not appear in ApoE^−/−^/gal-3^−/−^ mice [53]. Hsu et al. also demonstrated that lymphocyte amounts and macrophage infiltration ability decreased significantly in Gal-3 knockout mice compared to wild-type mice stimulated by inflammation [54]. These studies indicated that Gal-3 may exacerbate atherosclerosis through the activation of inflammation. In addition, phagocytosis of erythrocytes by macrophages has been regarded as a major pathological change in the progression of atherosclerosis. Sano et al. have investigated that the Gal-3-deficient macrophages reduced phagocytosis of erythrocytes and apoptotic thymocytes *in vitro* and *in vivo*, so Gal-3 may promote the phagocytosis of erythrocytes to aggravate atherosclerosis [55]. Arar et al. have demonstrated that the expression of LGALS3 was inactivated in quiescent vascular smooth muscle cells, but activated significantly in the aortas of hypercholesterolemic rabbits, balloon-injured rats, and cultured smooth muscle cells [56].

In recent years, some research has concentrated on atherosclerosis associated with heart failure and the mechanism that involves Gal-3. Yu et al. indicated that collagen production, processing, cleavage, cross-linking, and deposition were downregulated in Gal-3 knockout mice compared to those in wild-type mice. Moreover, cardiac remodeling, cardiac fibrosis, left ventricular dysfunction, and heart failure development were also found to be attenuated in Gal-3 knockout mice [57]. In the study by Watson et al., concomitant inhibition of renin-angiotensin system (RAS) and RAGE attenuated the development of atherosclerosis significantly. RAGE^−/−^/ApoE^−/−^ mice had less plaque area and attenuated macrophage infiltration than ApoE^−/−^ mice [58]. Gal-3 is regarded as a type of RAGE, so the concomitant inhibition of RAS and Gal-3 may be a potential strategy for the treatment of atherosclerosis.

However, there are a few studies that demonstrate that Gal-3 is protective for atherosclerosis. Iacobini et al. indicated that the atherosclerosis plaque area of Gal-3 knockout mice was higher than that of wild-type mice, and increased accumulation of oxidized low-density lipoprotein was observed in Gal-3 knockout mice [59]. A study also found that the macrophage infiltration and markers of systemic inflammation increased in LGALS3^−/−^ mice compared with wild type [60]. Gal-3 may not only have negative effects but also have some positive effects on atherosclerosis. Hence, the effect of Gal-3 in atherosclerosis deserves further examination.

## 5. Epidemiological Studies of Gal-3 in Atherosclerosis

### 5.1. Gal-3 and Atherosclerosis

As a new biomarker of cardiovascular disease, the role of Gal-3 has been investigated by substantial epidemiological research. Several studies [61, 62] have suggested that the measurement of plasma Gal-3 concentration may be a good biomarker of diseases related to atherosclerosis. Nevertheless, the epidemiological studies associated with Gal-3 and atherosclerosis have primarily concentrated on heart failure and coronary artery disease in the last several years. Gal-3 was an independent risk factor for cardiovascular disease. A 10-year cohort study of 7968 participants indicated that the population with high Gal-3 levels was prone to suffer from cardiovascular disease [63]. Elderly individuals (mean age 69 years) with low Gal-3 had remarkably low cardiovascular risk over the follow-up period of 2.7 years [64]. Madrigal-Matute et al. indicated that Gal-3 plasma level was positively associated with carotid intima-media thickness and Gal-3 level increased in patients with carotid atherosclerosis compared with healthy controls [29]. Pusuroglu et al. and Ozturk et al. found that Gal-3 increased as carotid atherosclerosis became heavier in specific populations including obstructive sleep apnea syndrome and type 2 diabetes mellitus patients [65, 66]. Anyfanti et al. recently declared that in a cohort of relatively well-controlled rheumatoid arthritis (RA) patients with long-standing disease and low levels of systemic inflammation, serum Gal-3 levels were positively associated in the univariate analysis with carotid intima-media thickness, the marker of subclinical atherosclerosis, suggesting the potential utility of Gal-3 as a biomarker for subclinical cardiovascular disease in patients with RA [67]. Gal-3 is also shown to be a significant and independent predictor for coronary atherosclerosis [68]. In addition, Pei et al. investigated the effect of berberine on ox-LDL-induced macrophage activation and Gal-3 expression in neoatherosclerosis patients after percutaneous coronary intervention. They indicated that berberine suppressed Gal-3 upregulation and ox-LDL endocytosis through the NF-*κ*B and AMPK signaling pathways [69]. These studies showed a potential property of Gal-3 as a biomarker for atherosclerosis.

### 5.2. Gal-3 and Heart Failure

Chronic heart failure is a series of complex syndromes associated with disability of cardiac ventricular ejection caused by organic or functional heart disease [70–72]. Current clinical biomarkers of chronic heart failure are brain natriuretic peptide and N-terminal pro-brain natriuretic peptide that have some limitations including age, renal function, and obesity [73–75]. Thus, searching for a new effective biomarker for heart failure is needed urgently. In the DEAL-HF study, 232 chronic heart failure patients were examined and followed up for four years. The baseline Gal-3 level was significantly associated with the prognosis and mortality of patients [76]. A study from de Boer et al., including 592 chronic heart failure patients whose left ventricular ejection fraction was less than 35%, also showed a consistent result with the DEAL-HF study. They indicated that Gal-3 was an independent marker for the prognosis of heart failure patients [77]. The Gal-3 level in chronic heart failure patients was higher than that in healthy controls, and increased Gal-3 was associated with the severity of heart failure and its complication as discussed by Meijers et al. [78]. Moreover, a Chinese population study indicated that the sensitivity and specificity of Gal-3 for heart failure diagnosis were 94.3% and 65.1%, respectively, as Gal-3 concentration reached 17.8 ng/mL [79]. In the CORONA study, 1492 patients with heart failure caused by ischemic myocardiosis were randomly divided into a Rosuvastatin treatment group and a control group: the mortality of the statin group was lower than that of the control group and Gal-3 decreased significantly in the statin group [80]. Polat et al. found that Gal-3 played an important role in prevention, classification, and personal therapy of heart failure [81]. Aforementioned studies revealed the clinical significance of Gal-3 in atherosclerosis-associated heart failure, but the mechanism of Gal-3 leading to heart failure needs to be further detected.

### 5.3. Gal-3 and Coronary Heart Disease

Except for heart failure, a great number of studies found that Gal-3 could also be regarded as an effective biomarker for coronary heart disease. Falcone's study included 125 coronary artery disease patients which were categorized into two groups with unstable angina or stable angina. Gal-3 level was significantly higher in unstable angina than stable angina, and it was higher in patients with three pieces of coronary artery disease than with one-two pieces [82]. Higueras et al. also indicated that Gal-3 was a risk factor for unstable angina [83]. Another study indicated that elevated circulating level of Gal-3 could be regarded as an independent predictor of the combined 30-day major adverse clinical outcome in patients with ST-segment elevation myocardial infarction undergoing primary percutaneous coronary intervention [84]. In a community cohort of patients with incident myocardial infarction (MI), elevated Gal-3 remained associated with increased risk of mortality and heart failure after adjustment for age, sex, comorbidities, and troponin, suggesting a role for measuring Gal-3 levels as a risk evaluation post-MI [85]. Moreover, Szadkowska et al. indicated that Gal-3 was an independent risk factor for reinfarction in MI patients after interventional operation, as Gal-3 reached 18.1 ng/mL [86]. In a prospective study containing 782 patients with coronary heart disease, the prognosis of low-Gal-3 patients was better than that of high-Gal-3 patients [87]. Moreover, the combined detection of Gal-3 and carotid intima-media thickness has been verified to be a more effective prediction for coronary artery disease [88]. Grandin et al. found that the acute coronary syndrome patients with high Gal-3 levels were more prone to suffer heart failure than low-Gal-3 patients [89]. Here, we summarized genetic and epidemiological studies of Gal-3 associated with atherosclerosis over the past two decades (Table 1).

## 6. Gal-3 Modulation

Gal-3 inhibition might be beneficial for atherosclerosis treatment due to the important role of Gal-3 in atherosclerosis and atherosclerosis-associated heart failure. As Gal-3 recognizes and binds to glycoprotein oligosaccharides via its carbohydrate recognition region, pharmacological inhibition of Gal-3 has almost exclusively targeted the CRD for inhibiting activities of this protein. Modified citrus pectin (MCP), a natural polysaccharide extracted from citrus plants, has been applied in experimental studies as a classical Gal-3 inhibitor. Lu et al. found that MCP inhibited the adhesion of leucocytes to endothelial cells to relieve atherosclerotic lesions through Gal-3 inhibition [90]. MacKinnon et al. also indicated that administration of MCP reduced the atherosclerosis plaque volume in ApoE^−/−^ mice [52]. Gal-3 inhibition induced by MCP could also reverse the isoproterenol-induced left ventricular dysfunction characterized by reducing myocardial inflammation and fibrogenesis in heart failure mice [91]. Thiodigalactoside (TDG) derivatives targeting CRD sites have been approved as antagonists of Gal-3. TD-139, a thiodigalactoside analogue, has been used as an inhaled powder for the treatment of idiopathic pulmonary fibrosis and is speculated to antagonize Gal-3 [92]. Multivalent attachment of a TDG derivative using the bovine serum albumin has been identified as one of the most potent Gal-3 inhibitors so far [93].

At present, the frequently used clinical drug associated with Gal-3 to treat cardiovascular disease is statin. Cannon et al. indicated that the acute coronary syndrome patients with statin drug treatment had a lower level of Gal-3 than patients with standardized treatment [94]. The CORONA study showed that Gal-3 level and all-cause mortality of Rosuvastatin-treated heart failure patients decreased significantly compared to the control group [80]. In addition, Lee et al. found that Atorvastatin markedly reduced Gal-3 expression within atherosclerotic plaques [46]. Moreover, some new Gal-3 inhibitors have been discovered in recent years. Watson et al. indicated that Quinapril treatment decreased Gal-3 expression, macrophage infiltration, and vascular collagen deposition within atherosclerosis plaque [58]. Lax et al. [95] demonstrated that mineralocorticoid receptor antagonists (MRAs) could inhibit Gal-3 to attenuate cardiac fibrosis, left ventricular dysfunction, and subsequent heart failure. Similarly, N-acetyllactosamine (LacNac) had the same effect with MRAs [57].

Regarding Gal-3 agonists, experimental evidence from Lin et al. revealed that aldosterone increased Gal-3 secretion in macrophages [96]. Another study from Qian et al. indicated that doxazosin exacerbated Gal-3 expression in cardiomyocytes [97]. The application of the aldosterone antagonist decreased the Gal-3 level and inhibited left ventricular dysfunction in patients with hypertension [98]. These findings proved that Gal-3 agonist inactivation may be useful for the clinical treatment of atherosclerosis (shown in Table 2).

## 7. Perspectives and Conclusions

There is strong evidence that Gal-3 participates in the initiation and progression of atherosclerosis. Numerous studies have shown that Gal-3 contributes to macrophage differentiation, foam cell formation, endothelial dysfunction, and VSMC proliferation and migration in atherosclerosis. In this review, we summarized several mechanisms pivotal to the development of atherosclerosis that are stimulated by local or circulating Gal-3. Amplification of cardiovascular inflammation and lipid accumulation in macrophage by Gal-3 are the most important mechanisms. Up to now, studies on Gal-3 have been focused on genetics and epidemiology suggesting that Gal-3 is positively related to the occurrence of atherosclerosis. Thus, Gal-3 can be regarded as a new biomarker for the risk evaluation of atherosclerosis and a new treatment target for atherosclerosis therapy. On the other hand, the application of Gal-3 inhibitors may be a potential treatment for atherosclerosis in the future. Moreover, macrophage plays a vital role in atherosclerosis, and some studies have indicated that Gal-3 can activate M2 macrophage differentiation, which has an anti-inflammatory property, through the CD98/phosphoinositide 3-kinase (PI3K) pathway [28, 99]. Activation of M2 macrophage differentiation by Gal-3 may be beneficial to the treatment of atherosclerosis through suppression of inflammation. As a key protein in autophagy progression, PI3K can be upregulated by Gal-3. Therefore, the augmentation of macrophage autophagy in atherosclerotic plaque through the Gal-3/PI3K/Akt/mTOR pathway is probably an effective therapy for atherosclerosis. To some extent, the multiple functions of Gal-3 depend on its N-terminal domain modification by matrix metalloproteinase (MMP). MMP-7 has been demonstrated to be a Gal-3 activator to exacerbate inflammation [100]. Hence, MMP can be regarded as a new target for further research on the connection between Gal-3 and atherosclerosis.

## Figures and Tables

**Table 1:** Brief research development of Gal-3 associated with atherosclerosis.

Research type	Targets	Main findings
Genetic	ApoE^−/−^/gal-3^−/−^ mice [52]	Atherosclerosis plaques were significantly smaller.
	ApoE^−/−^/gal-3^−/−^ mice [53]	Aortic atherosclerosis plaques decreased and vascular adventitia inflammation reduced.
	Gal-3 knockout mice [54]	Lymphocyte amounts and macrophage infiltration decreased significantly.
	Gal-3-deficient macrophages [55]	Phagocytosis of erythrocytes reduced.

Epidemiological	Heart failure patients [76]	Gal-3 level was significantly associated with the prognosis and mortality.
	Coronary heart disease patients [82]	Gal-3 level increased significantly in the unstable angina group.
	Myocardial infarction patients [86]	Gal-3 was regarded as an independent risk factor for reinfarction.
	Carotid atherosclerosis patients [29]	Gal-3 level was positively associated with carotid intima-media thickness and the prevalence of carotid atherosclerosis.
	Heart failure patients [79]	The sensitivity and specificity of Gal-3 for heart failure diagnosis were 94.3% and 65.1%, respectively.
	Patients with coronary artery disease [68]	Gal-3 was a significant and independent predictor.
	Patients with myocardial infarction [85]	Elevated Gal-3 was associated with mortality and heart failure.

**Table 2:** Classical Gal-3 modulators in the present relative studies.

Modulator	Subtype	Function	Targets	Mechanism	Authors
MCP	—	Inhibition	Leukocytes and endothelial cells	Inhibition of the adhesion between leucocytes and endothelial cells	Lu et al. [90]
			ApoE^−/−^ mice	—	MacKinnon et al. [52]
			Heart failure mice	Reducing myocardial inflammation and fibrogenesis	Vergaro et al. [91]

Statin	Pravastatin	Inhibition	Acute coronary syndrome patients	—	Cannon et al. [94]
	Rosuvastatin		Heart failure patients		Gullestad et al. [80]
	Atorvastatin		ApoE^−/−^ mice		Lee et al. [46]

Quinapril	—	Inhibition	Diabetic RAGE^−/−^/gal-3^−/−^ mice	Reducing macrophage infiltration and vascular collagen deposition	Watson et al. [58]
MRAs	—	Inhibition	Myocardial infarction mice	Attenuation of cardiac fibrosis, left ventricular dysfunction, and heart failure	Lax et al. [95]
LacNac	—	Inhibition	Gal-3^−/−^ mice		Yu et al. [57]
Aldosterone	—	Activation	Macrophages	—	Lin et al. [96]
Doxazosin	—	Activation	Cardiomyocytes	—	Qian et al. [97]

Abbreviations: Gal-3: galectin-3; MCP: modified citrus pectin; MRAs: mineralocorticoid receptor antagonists; LacNac: N-acetyllactosamine.
